# Cadmium-Related Effects on Cellular Immunity Comprises Altered Metabolism in Earthworm Coelomocytes

**DOI:** 10.3390/ijms21020599

**Published:** 2020-01-17

**Authors:** Martina Höckner, Claudio Adriano Piechnik, Birgit Fiechtner, Birgit Weinberger, Lars Tomanek

**Affiliations:** 1 Institute of Zoology, Center for Molecular Biosciences Innsbruck, University of Innsbruck, 25 6020 Technikerstraße, Austria; birgit.fiechtner@uibk.ac.at; 2 Department of Cell Biology, Adaptive Biology Laboratory, Universidade Federal do Paraná, Curitiba, Paraná 82590-300, Brazil; claudio.piechnik@gmail.com; 3 Institute for Biomedical Aging Research, University of Innsbruck, 6020 Innsbruck, Austria; birgit.weinberger@uibk.ac.at; 4 Environmental Proteomics Laboratory, Department of Biological Sciences, Center for Coastal Marine Studies, California Polytechnic State University, 1 Grand Ave., San Luis Obispo, CA 93407-0401, USA; ltomanek@calpoly.edu

**Keywords:** coelomocytes, earthworm, metabolism

## Abstract

The heavy metal cadmium (Cd) is known to modulate the immune system, challenging soil-dwelling organisms where environmental Cd pollution is high. Since earthworms lack adaptive immunity, we determined Cd-related effects on coelomocytes, the cellular part of innate immunity, which is also the site of detoxification processes. A proteomics approach revealed a set of immunity-related proteins as well as gene products involved in energy metabolism changing in earthworms in response to Cd exposure. Based on these results, we conducted extracellular flux measurements of oxygen and acidification to reveal the effect of Cd on coelomocyte metabolism. We observed a significantly changing oxygen consumption rate, extracellular acidification, as well as metabolic potential, which can be defined as the response to an induced energy demand. Acute changes in intracellular calcium levels were also observed, indicating impaired coelomocyte activation. Lysosomes, the cell protein recycling center, and mitochondrial parameters did not change. Taken together, we were able to characterize coelomocyte metabolism to reveal a potential link to an impaired immune system upon Cd exposure.

## 1. Introduction

The earthworm immune system consists of cellular and humoral factors that are all part of innate immunity, as earthworms lack an adaptive immune system. However, it has to be mentioned that borders between innate and adaptive immunity are fluid, revealing many categories in between [1,2]. So-called coelomocytes execute the functions of cellular immunity in earthworms [3]. They are found in the coelomic fluid of the body cavity and are released through dorsal pores to fight pathogens and help cope with environmental stress. Coelomocytes are comprised of different cell types like amoebocytes and eleocytes. Amoebocytes are phagocytes, involved in reactive oxygen species (ROS) production that possess natural killer (NK) cell-like activity and express Toll-like receptors. Eleocytes maintain the pH of the coelomic fluid, and store glycogen, lipids and, moreover, riboflavin [4]. Riboflavin, the homolog of mammalian vitamin B2, acts as antinociceptive, anti-inflammatory agent, and chemoattractant [5]. Importantly, coelomocyte activity is mediated via calcium, which is, depending on the earthworm species and coelomocyte subtype, released from intracellular calcium stores and/or increased by calcium influx [6].

Coelomocytes are specific sites of the cellular stress response, which includes the expression of metallothioneins (MTs) [5]. MTs can bind heavy metals like Cadmium (Cd) to their cysteine residues. The heavy metal protein complex is then stored in lysosomes as shown in the mouse kidney [7]. Cd is classified as a carcinogen by the WHO and is brought into the environment mainly through industry and agriculture. The chemical and structural similarity of Cd to calcium and zinc, which is called ionic mimicry, is the main pathway by which Cd exerts its toxicity [8]. Therefore, soil-dwelling organisms like earthworms face challenging conditions in Cd-polluted habitats. 

In general, Cd causes coelomocyte numbers to decrease in earthworms [5,9]. Cd exposure also leads to a decreased number of hemocytes in a clam species [10] and in freshwater crab [11]. Cd showed immunosuppressive effects in mammals [12], several fish species [13,14,15,16], amphibians [17] and invertebrates [10,11]. A study in mouse monocytes, which are phagocytes of the innate immune system, suggested that Cd-induced ROS production leads to ER stress and calcium-mediated calpain activation that in turn triggers autophagy and apoptosis via caspases [12]. Caspase 3, a mediator of cell death, was activated in earthworm coelomocytes upon Cd exposure [9]. 

Cd treatment also leads to lysosomal membrane permeabilization [18] and a decrease of acidic organelles in hemocytes of the zebra mussel [19]. Quite recently, the lysosome function was extended from just being the cell’s “trash can” to an important regulator of cellular metabolism [20]. The authors of the latter study suggested that lysosomes control the turnover of metabolic building blocks, e.g., amino acids, and communicate the metabolic state of the cell through so-called nutrient sensing modules. 

What is still largely unknown are the mechanisms behind Cd-induced immunomodulatory effects. A proteomic study on Cd-exposed earthworms provided data on several functional protein classes, like immunity, calcium signaling, and energy metabolism. Therefore, we focus on the metabolic profile of earthworm immune cells when exposed to Cd in vivo and in vitro to reveal the functional context of Cd-induced immunodeficiency and energy homeostasis. 

## 2. Results

After exposure of earthworms to 50 mg CdCl_2_/ kg dry soil for 2 weeks, a proteomics approach revealed that proteins from several functional classes changed significantly compared to controls. 

**Figure 1:**
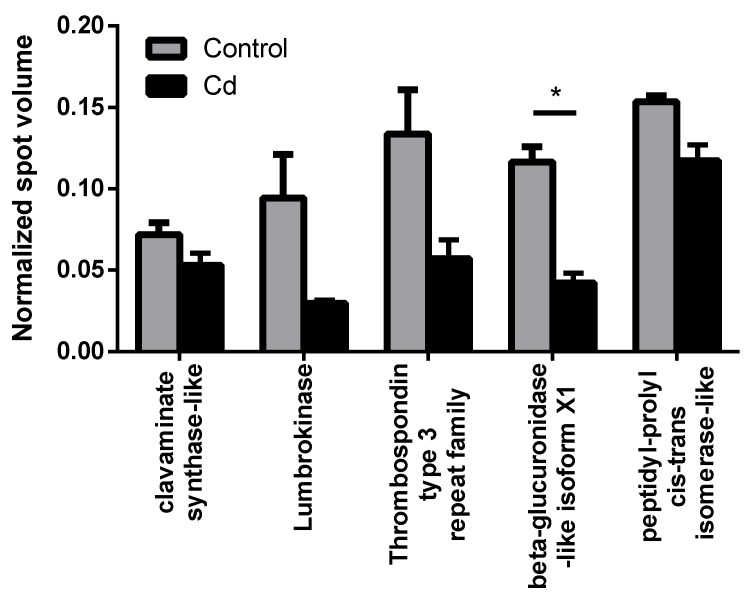
Immunity-related proteins, which were detected in the proteomics approach in controls and after two weeks of Cd exposure (50 mg/kg dry soil). * *t*-test indicates significant differences at *p* ≤ 0.05.

**Figure 2:**
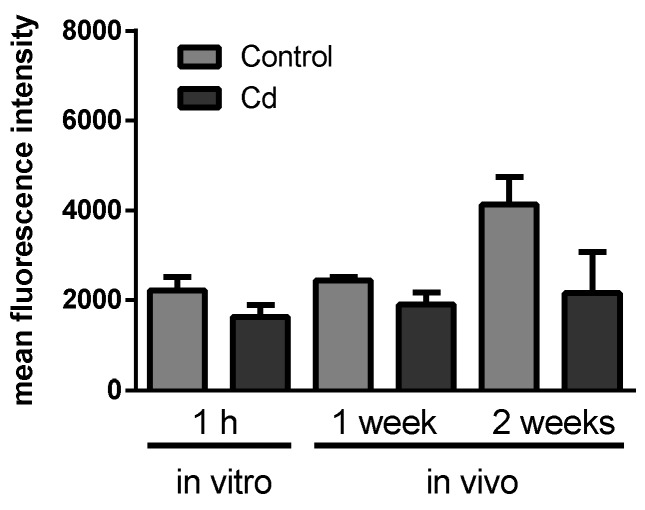
Flow cytometry of coelomocytes derived from Cd-exposed (50 mg/kg dry soil) and control earthworms stained with LysoTracker^®^ Green to determine lysosome numbers. In vitro exposures were accomplished using 200 µM CdCl2. *t*-tests did not indicate any significant differences.

We observed the most prominent results in immunity-related proteins, which all showed less abundance in the Cd exposure group. Due to high individual variability, only β-glucuronidase revealed a statistically significant decrease (Figure 1). We detected a Lumbrokinase protein, which is a fibrinolytic enzyme [21], that was lower in abundance with Cd exposure. Hypercoagulability has already been shown to occur in women with myeloma and endometrial cancer in response to Cd exposure [22]. We found a thrombospondin type 3 repeat family protein, which is a mediator of the inflammatory response. Thrombospondin increased in rats after 8 h of a hepatotoxic dose of Cd [23], which could, however, be a time-dependent effect. We identified a clavaminate synthase (CAS)-like gene product, which belonged to the non-heme iron, α-ketoglutarate-dependent oxygenase, and was responsible for the production of antibiotic compounds [24]. β-glucuronidase was observed in basophils and neutrophils of earthworm coelomocytes in vesicles, which were suggested to be lysosomes [25]. As a consequence, we measured the number of lysosomes, since reduced levels of β-glucuronidase could reflect a decreasing lysosome number [26]. Although a slight trend of decreasing lysosome numbers was observed, flow cytometry experiments did not confirm significant differences in coelomocytes derived from Cd-exposed earthworms (Figure 2). 

**Figure 3:**
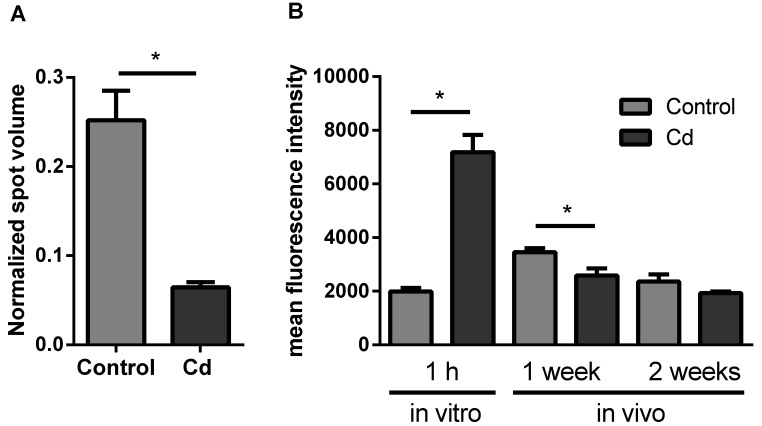
(**A**) Protein expression of Neuronal Calcium Sensor 2. Data derived from the proteomics approach. (**B**) Flow cytometric measurements to determine the intracellular calcium level using Fluo-3-am in coelomocytes from in vitro and vivo experiments. CdCl_2_ concentration in the soil was 50 mg/kg dry soil. * *t*-test indicates significant differences at *p* ≤ 0.05.

The proteomics approach revealed a significant change of neuronal calcium sensor 2, a protein involved in calcium signaling (Figure 3A). Intracellular calcium levels did not significantly change after 2 weeks of earthworm exposure in Cd-spiked soil, in contrast to a significant decrease after 1 week. In vitro Cd exposure for 1 h revealed a significant increase of intracellular calcium levels (Figure 3B). 

**Figure 4:**
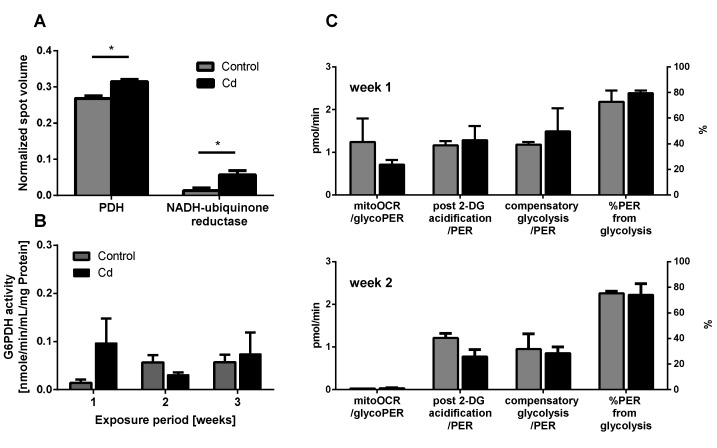
Energy metabolism. (**A**) Proteins related to energy metabolism detected in the proteomics approach. (**B**) Glucose-6-Phosphate Dehydrogenase (G6PDH) activity measurement using coelomocytes derived from control and Cd-exposed earthworms. (**C**) Glycolytic rate assay using coelomocytes from earthworms exposed to control or Cd-spiked soil (50 mg/kg dry soil). * *t*-test indicates significant differences at *p* ≤ 0.05.

Several proteins involved in energy metabolism revealed a statistically significant increase in abundance upon Cd exposure (Figure 4A). Higher expression levels of pyruvate dehydrogenase (PDH) hint towards an increasing production of acetyl-CoA, which feeds into the citric acid cycle for energy and substrate production. The detection of increasing levels of NADH ubiquinone reductase (NADH dehydrogenase), the first enzyme of the electron transport chain further confirmed the potential increase in aerobic metabolism (Figure 4A). 

**Figure 5:**
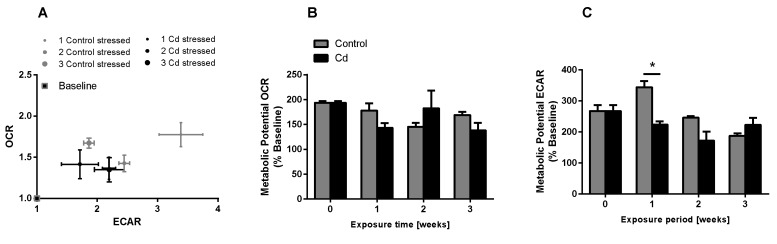
Energy phenotype assay of coelomocytes derived from control and Cd-exposed earthworms (50 mg/kg dry soil). (**A**) Oxygen consumption rate (OCR) vs extracellular acidification rate (ECAR). The term stressed refers to the state after Oligo/FCCP addition. (**B**,**C**) Metabolic ECAR and OCR potential. * ANOVA and multiple comparisons of Cd treatment using the Tukey test indicates significant differences at *p* ≤ 0.05.

**Figure 6:**
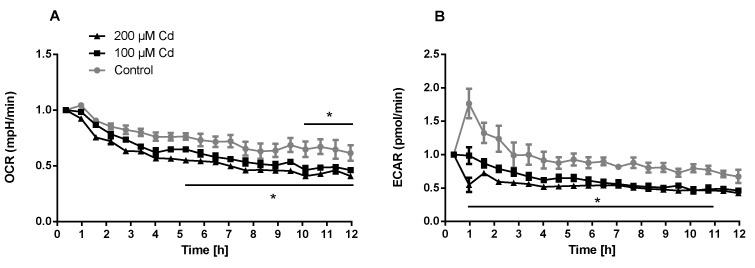
Extracellular flux measurements of oxygen (OCR) and protons (ECAR) of coelomocytes exposed in vitro to different concentrations of Cd. * Two-way ANOVA and Tukey’s multiple comparisons test indicates statistical significance at *p* ≤ 0.05.

**Figure 7:**
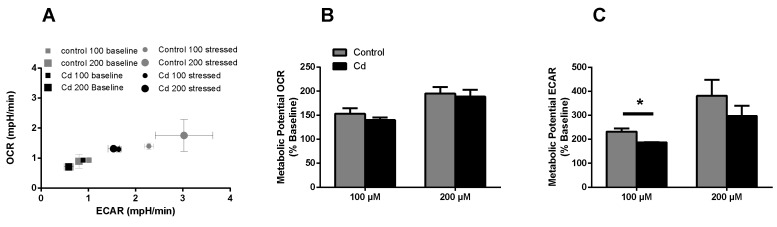
Energy phenotype assay (in vitro). (**A**) OCR vs ECAR of control and Cd-exposed coelomocytes. The term stressed refers to the state after Oligo/FCCP addition. (**B**,**C**) Metabolic ECAR and OCR potential of in vitro Cd exposed coelomocytes. * ANOVA on ranks and post-hoc Tukey test revealed significant differences at *p* ≤ 0.05.

**Figure 8:**
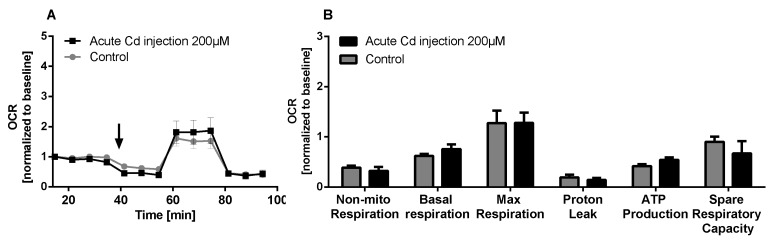
Mito stress test of coelomocytes. (**A**) OCR of coelomocytes including an acute injection of 200 µM CdCl2 indicated by the arrow. (**B**) Mito stress test parameters. ANOVA did not detect any significant differences.

The immune system, as well as energy metabolism, is affected by Cd. We therefore used coelomocytes, earthworm immune cells, to measure oxygen consumption and extracellular acidification after in vivo Cd exposure. Whether a shift of glucose into the pentose phosphate pathway (PPP) can lead to decreased extracellular acidification was tested by measuring Glucose-6-Phosphate Dehydrogenase (G6PDH) activity, which, however, did not reveal a significant increase in coelomocytes derived from earthworms exposed to Cd (Figure 4B). We showed that around 70% of total extracellular acidification in coelomocytes derives from glycolysis, and this did not change after Cd exposure, similar to the other parameters measured in the glycolytic rate assay (Figure 4C). The metabolic potential describes the maximum ability to meet an energy demand via mitochondrial respiration and glycolysis. The metabolic potential can also be referred to as the response to an induced energy demand. Both the oxygen consumption rate (OCR) (Figure 5B) and extracellular acidification rate (ECAR) (Figure 5C) of this induced (stressed) state were measured. In coelomocytes derived from Cd-exposed earthworms, the OCR metabolic potential did not significantly change, in contrast to the metabolic potential, as indicated by the ECAR (Figure 5). The timeline of OCR and ECAR before and after the addition of the uncoupling agent FCCP and oligomycin—an ATPase inhibitor—are given in Supplementary Figure S1 When measuring the OCR and ECAR over a period of 12 h, Cd caused decreased rates in both cases (Figure 6). In vitro Cd exposure experiments of coelomocytes confirmed the results from the in vivo studies, namely that Cd leads to a decreased metabolic potential regarding ECAR. The same as in the in vivo experiments, the OCR metabolic potential did not significantly change (Figure 7). Details of the in vitro energy phenotype test of coelomocytes regarding OCR and ECAR are shown in Supplementary Figure S2. Furthermore, we characterized coelomocytes according to their oxygen consumption and calculated aerobic capacity, ATP production, proton leak, maximum mitochondrial respiration and non-mitochondrial respiration. Aerobic capacity can be calculated by subtracting the basal respiration from maximum respiration (Figure S3). Inhibition of mitochondrial function by Rotenone and Antimycin A (Rot/AA) enables calculation of mitochondrial-associated acidification (Figure S3). In vitro coelomocyte exposure to Cd revealed no significant changes (Figure 8).

## 3. Discussion

The present study focuses on coelomocytes to study the response to Cd on the innate immune system and energy metabolism. It is already known that the heavy metal Cd affects the immune system, but the actual biochemical and physiological mechanisms of these effects are largely unexplored. A proteomics approach identified several proteins related to immunity, confirming the effect on the immune system, and revealing a slight down-regulation when earthworms were exposed to Cd for two weeks. In contrast, proteins involved in energy metabolism showed a Cd-dependent increase. We suggest that higher levels of PDH and NADH dehydrogenase hint towards an increased energy demand due to the activation of stress-responsive mechanisms for heavy metal detoxification and for scavenging reactive oxygen species (ROS) in earthworm tissue [27] and coelomocytes [4]. A previous study on earthworms showed that lipid storage was primarily depleted due to Cd, followed by complete consumption of carbohydrates [28], supporting our conclusion that Cd exposure is energetically expensive. 

We focused on the analysis of coelomocytes, to reveal mechanistic details of an impaired immune system and altered energy metabolism.

The metabolic ECAR potential of coelomocytes that derived from Cd-challenged earthworms decreased (determined by measuring ECAR after inhibition of ATP synthase and decoupling of the proton gradient using oligomycin and FCCP, respectively). This indicates that the response to an induced energy demand is impaired and may limit an organism’s ability to cope with any additional environmental stress. We conclude that immune cells of Cd-exposed earthworms perform worse than controls in case of an acute requirement of energy resources. Cd did not, on the other hand, affect the metabolic potential of oxygen consumption rate (metabolic OCR potential). We conclude from these results that Cd interferes with the anaerobic rather than the aerobic branch of cellular metabolism during an induced energy demand. Results from in vitro exposure experiments of coelomocytes without the use of inhibitors showed reduced oxygen consumption and reduced extracellular acidification due to Cd in a time- and dose-dependent manner. 

In general, extracellular acidification results from the production of lactate in glycolysis, the production of CO_2_ in the citric acid cycle followed by the reaction with water to bicarbonate and protons, but also oxidative phosphorylation in the mitochondria leads to the release of protons. We found that around 70% of acidification results from glycolysis in coelomocytes. We therefore assumed that the decreased metabolic ECAR potential upon Cd exposure is due to Cd-based interference with glycolysis. However, when we measured the glycolytic rate in coelomocytes from control or Cd-exposed earthworms, we did not discover a difference. To investigate whether the PPP plays a role in altering extracellular acidification, we determined the activity of Glucose-6-Phosphate Dehydrogenase, which did not change in the Cd exposure groups. The PPP is involved in producing reduction equivalents during Cd exposure. G6PDH activity increased in the hepatopancreas and ovary upon Cd exposure in a freshwater crab in a time-dependent manner and the produced reduction equivalents were used to recycle GSH [29]. This may suggest that the PPP is not a limiting factor for the production of reducing agents to scavenge ROS in earthworms. 

We did not observe differences in the glycolytic rate, and there was no change of G6PDH activity. We know from the literature that Cd affects malate and succinate in the TCA cycle, which should, however, not affect acidification, since CO_2_ is produced by alpha-ketoglutarate dehydrogenase. Previous studies have shown an inhibitory effect of Cd on LDH in rats [30]. LDH catalyzes the reaction of pyruvate to lactate and protons. Inhibition of LDH could indeed lead to reduced levels of protons. LDH is also responsible for lysosome acidification in cancer cells [31]. 

Quite recently, lysosomes have been addressed as a master regulator of cellular energy metabolism via the mTOR pathway [20]. In human endothelial cells, Cd-induced lysosomal disruption has been studied as part of the autophagy signaling [18]. The proteomics approach revealed that peptidyl-prolyl cis-trans isomerase like protein (PPI) slightly decreased upon Cd exposure. PPIs are involved in regulation of the mTOR pathway and of immunosuppressive reactions [32]. However, we could not show that the number of lysosomes changed in coelomocytes upon Cd treatment. Cd-induced inhibition of mTOR has been shown previously leading to autophagy, suggesting that autophagy plays an important role in Cd-induced immune deficiency [12]. Independent of the lysosome number, the lysosomal function might be impaired, which has, however, not been investigated herein.

Calcium mediates the activation of coelomocytes [6] and it has been shown to be involved in the response to Cd in yeast [33]. In freshwater crab, Cd induces apoptosis via calcium signaling [34]. We found an acute effect of Cd on the intracellular calcium level in coelomocytes, which increased after 1h of exposure, suggesting that calcium plays a role in the acute response to Cd and probably immune cell activation. Interestingly, when we looked at coelomocytes derived from 1 week Cd-exposed earthworms, we observed a decrease in [Ca_2+_]_I_, which was still slightly but not significantly reduced after two weeks of Cd exposure. Calcium signaling might therefore play an important role in Cd-dependent immune deficiency.

When we measured parameters of mitochondrial metabolism in coelomocytes, we did not find any Cd-dependent effects. We observed around 30% proton leak (in relation to basal respiration). The expression of uncoupling proteins (UCPs) is responsible for decoupling of the proton gradient in mitochondria. In the past years the crucial role of UCPs in redox signaling of immune cells has been indicated [35]. Activation of UCP2, and the resulting proton leak, negatively regulates ROS formation and ROS signaling [35]. In contrast to results found in the present study, Cd has been shown to lead to partial uncoupling of the proton gradient in oyster mitochondria [36]. Cd exposure also increases proton conductance in the hemocytes of clams [37]. 

## 4. Materials and Methods 

### 4.1. Earthworm Maintenance

For the proteomics approach, the earthworm species *L. rubellus* was used due to the availability of the *L. rubellus* genome (Supplementary Material Section S1). However, since only *L. terrestris* is commercially available, further studies were carried out using this species, which was ordered from Wurmwelten.de (Stadtoldendorf, Germany). Earthworms for coelomocyte harvesting were kept in tanks containing commercially available feeding soil (Wurmwelten.de, Stadtoldendorf, Germany) at 15 °C with a 12/12 light/dark cycle. The soil used for the acclimation and exposure was heat-treated (120 °C, 12 h). A water content of 50% was maintained, and the earthworms were fed weekly with horse manure (1.2 g manure per individual). Following 4 weeks of acclimation, earthworms were exposed either to control soil or to 50 mg/kg CdCl_2_ for 3 weeks. The exposure concentration was chosen according to an environmentally relevant impact of Cd in highly polluted areas [38].

### 4.2. Proteomics Approach

In short, proteins were isolated from the whole tissue parts of the posterior region behind the clitellum, separated using 2D gel electrophoresis, and stained with colloidal Coomassie Blue reagent following published protocols [39]. The proteome map was analyzed using Delta2D software (version 4.3; Decodon, Greifswald, Germany) and significantly changed protein spots were then picked and analyzed using MALDI-MS/MS. Proteins were subsequently identified by blasting against a *L. rubellus* genome database (http://badger.bio.ed.ac.uk/earthworm/, last access date: 18 November 2013; this site has closed down since the data analysis was completed). The control and Cd-exposed group consisted each of three biological replicates. Detailed information on the proteomics approach can be found in the Supplementary Material, Section S1.

### 4.3. Harvesting of Coelomocytes

Earthworms (*L. terrestris*) were washed in water to remove excess soil and placed into a petri dish containing 1xPBS including 50 mM guaiacol glyceryl ether (GGE) (extrusion buffer). Earthworms were stimulated to extrude coelomocytes using a 9V battery and the cell suspension was centrifuged for 10 min at 1500 rpm at room temperature. Cells were resuspended in assay media (for details on the assay media please refer to the respective methodical sections).

### 4.4. Extracellular Flux Measurements

Coelomocytes were non-invasively harvested as described above. Cell number was measured with a Countess II automated cell counter (ThermoFisher, Waltham, MA, USA). Using the seahorse XFp analyzer (Agilent Technologies, Santa Clara, California, United States), the oxygen consumption rate (OCR) as well as the extracellular acidification rate (ECAR) was measured. 40,000 cells were seeded to cell cartridges in assay media as described in the user manual. For characterizing the metabolic potential, the Agilent Seahorse XFp Cell Energy Phenotype Test Kit was used according to manufacturer’s instructions. The metabolic potential can be defined as the response, e.g., the rate of increase or magnitude reached in metabolic rate, to an induced energy demand. Also, the glycolytic rate assay as well as the Mito stress test was applied for metabolic measurements (Agilent Technologies, Santa Clara, CA, USA). Data were normalized to the last measurement before application of inhibitors used in the respective assay to account for putative differences in cell numbers. Due to the complex nature of the assays, we provide more detailed information in the Supplementary Material (Section S3, Figures S3–S5).

### 4.5. Glucose-6 Phosphate Dehydrogenase Activity

G6PDH was measured using the glucose-6 phosphate dehydrogenase assay kit (Sigma-Aldrich, St. Louis, MO, USA) according to the user manual in coelomocytes derived from 1, 2, and 3 weeks Cd-exposed (50 mg CdCl_2_/kg dry soil) and control earthworms.

### 4.6. Lysosome Quantification

Coelomocytes were harvested from Cd-exposed and control earthworms after 1 and 2 weeks. Coelomocytes derived from control earthworms were also exposed to 200 µM CdCl2 for 1 h in vitro to measure the acute responses to Cd exposure. Cells were stained for 45 min with 1 mL LysoTracker^®^ Green DND-26 (Thermo Fisher Scientific, Waltham, MA, USA) at 15 °C. Fluorescence was detected by flow cytometry using a BD FACSCanto™ II instrument (Becton Dickinson, Franklin Lakes, NJ, USA). Coelomocyte subpopulations were not separately analyzed. A minimum of 10,000 cells were acquired in each experiment for flow cytometric analysis.

### 4.7. Calcium Measurements

Coelomocytes were harvested from Cd-exposed and control earthworms after 1 and 2 weeks (see above), and coelomocytes derived from control earthworms were also in vitro exposed to 200 µM CdCl_2_ for 1 h. Cells were stained with Fluo-3-am for 45 min at 15 °C (Sigma-Aldrich, St. Louis, MO, USA). Fluorescence was detected by flow cytometry using a BD FACSCanto™ II instrument (Becton Dickinson, Franklin Lakes, NJ, USA). Coelomocyte subpopulations were not separately analyzed. A minimum of 18,000 cells were acquired in each experiment for flow cytometric analysis.

### 4.8. Statistics

Statistical analyses were performed using SigmaPlot (version 14.0, Systat Software GmbH Erkrath, Germany). In cases where the data were normally distributed and passed the equal variance test, a *t*-test or ANOVA was conducted. In cases where normality or equal variance tests failed, a Mann–Whitney Rank Sum Test was applied. Multiple comparisons were performed using post-hoc Tukey tests. All data are presented as mean values ± SEM. 

## 5. Conclusions

Based on our results, we conclude that the effects of Cd leading to an impaired immune system in earthworms might in part be due to an altered metabolism, as well as impaired calcium signaling in coelomocytes. We could not show changing numbers of lysosomes in coelomocytes when earthworms were challenged with Cd and mitochondrial parameters also did not change upon acute Cd exposure. Further studies are needed to elucidate the specific mechanism behind compromised immunity due to Cd exposure.

We know from previous studies that the adverse effects of Cd are highly variable, so we assume that adverse effects of Cd are tissue-specific and largely time- and dose-dependent [40].

## Supplementary Materials

Supplementary materials can be found at https://www.mdpi.com/1422-0067/21/2/599/s1.

## Abbreviations

ATP	Adenosine triphosphate
[Ca_2+_]_I_	Intracellular Calcium concentration
Cd	Cadmium
CdCl_2_	Cadmiumchloride
CO_2_	Carbon dioxide
ECAR	Extracellular acidification rate
FCCP	Carbonyl cyanide-4-(trifluoromethoxy)phenylhydrazone
GGE	Guaiacol glyceryl ether
G6PDH	Glucose-6-phosphate dehydrogenase
LDH	Lactatedehydrogenase
MT	Metallothionein
NAD	Nicotinamide adenine dinucleotide
OCR	Oxygen consumption rate
PDH	Pyruvate dehydrogenase
PPP	Pentose phosphate pathway
PPI	Peptidyl-prolyl cis-trans isomerase-like protein
ROS	Reactive oxygen species
TCA	Tricarboxylic acid cycle
UCP	Uncoupling protein
