# Supplementary material for: Cadmium-Related Effects on Cellular Immunity Comprises Altered Metabolism in Earthworm Coelomocytes
Source: Int J Mol Sci. 2020 Jan 17;21(2):599. doi: 10.3390/ijms21020599 (PMC7013597; doi:10.3390/ijms21020599)
Supplement: Supplementary file 1 [file ijms-21-00599-s001.zip › ijms-689996-SI.pdf]

Supplementary Material

1. Proteomics

1.1. Animal collection and maintenance

L. rubellus was collected from a non-contaminated field site in Axams (Tirol, Austria) and brought to the lab to acclimate in soil that originated from the collection site. The worms were kept in plastic boxes at 15 °C with a 12/12 light dark cycle.

1.2. Experimental design

L. rubellus was used as study organisms to reveal proteomic differences upon Cd exposure. We used *L. rubellus* because of its susceptibility to metals and available genome and transcriptomic sequences. Control earthworms were exposed at 15 °C to control soil, Cd exposures were performed at 15 °C (50 mg/kg dry soil). 100 g dry soil / individual was used and five individuals were kept per box and experimental group, with a 12/12 light dark cycle. No mortality was observed during the whole exposure experiment. The earthworms were sampled after 14 days by cutting the distal part from behind the clitellum and shock-frozen in liquid nitrogen. The tissue samples were stored at −80 °C until further processing.

1.3. Homogenization

Frozen *L. rubellus* tissue was homogenized 1:4 in buffer [7mol L^{-1} urea, 2mol L^{-1} thiourea, 1% ASB-14 (amidosulfobetaine-14), 40mmol L^{-1} Tris-base, 0.5% immobilized pH 4–7 gradient (IPG) buffer (GE Healthcare, Piscataway, NJ, USA) and 40 mmolL^{-1} dithiothreitol], using an ice-cold ground-glass homogenizer. The homogenate was subsequently centrifuged at room temperature for 30 min at 16,100× *g*. Proteins of the supernatant were precipitated by adding four volumes of ice-cold 10% trichloroacetic acid in acetone and the solution was incubated at −20 °C overnight. After centrifugation at 4 °C for 15 min at 18,000× *g*, the supernatant was discarded and the remaining pellet was washed with ice-cold acetone, and centrifuged again before being re-suspended in rehydration buffer (7 mol L^{-1} urea, 2molL^{-1} thiourea, 2% CHAPS (cholamidopropyl-dimethylammonio-propanesulfonic acid), 2% NP-40 (nonyl phenoxylpolyethoxylethanol-40), 0.002% Bromophenol Blue, 0.5% IPG buffer and 100 mmolL^{-1} dithioerythritol)) through vortexing. The protein concentration was determined with the 2D Quant kit (GE Healthcare, UK), according to the manufacturer's instructions.

1.4. Two-dimensional gel electrophoresis

Proteins (400 µg) were loaded onto IPG strips (pH4–7, 11 cm; GE Healthcare, UK) for separation according to their isoelectric point (pI). We started the isoelectric focusing protocol with a passive rehydration step (5 h), followed by 12h of active rehydration (50 V, rapid mode), using an isoelectric focusing cell (BioRad, Hercules, CA, USA). The following protocol was used for the remainder of the run: 500 V for 1h (rapid mode), 1000V for 1h (linear mode), 8000V for 2.5 h (linear mode), 8000 V for 2.5 h (rapid mode), and 500 V (rapid mode)—as soon as that step started, the samples were removed and the strips were frozen at −80 °C. Frozen strips were thawed and incubated in equilibration buffer (375 mmol L^{-1} Tris-base, 6 mol L^{-1} urea, 30% glycerol, 2% SDS (sodium dodecyl sulfate) and 0.002% Bromophenol Blue)) for 15 min, first with 65 mmol L^{-1} dithiothreitol and second with 135 mmol L^{-1} iodoacetamide. IPG strips were placed on top of a 12% polyacrylamide gel with a 0.8% agarose solution containing Laemmli SDS electrophoresis (or running) buffer (25 mmol L^{-1} Tris- base, 192 mmol L^{-1} glycine and 0.1% SDS). Gels were run (Criterion Dodeca; BioRad, USA) at 200V for 55min with a recirculating water bath set at 10 °C. Gels were subsequently stained with colloidal Coomassie Blue (G-250) overnight and de-stained by washing repeatedly with Milli-Q (Millipore, Billerica, MA,

USA) water for 48h. The resulting gel images were scanned with a transparency scanner (model 1280; Epson, Long Beach, CA, USA).

1.5. Gel image analysis

Digitized images of two-dimensional gels were analyzed using Delta2D (version 4.3; Decodon, Greifswald, Germany) [7]. We used the group warping strategy to connect gel images through match vectors. Images of all treatments were fused into a composite image (proteome map), which represents mean volumes for each spot. Spot boundaries were detected within the proteome map and transferred back to all gel images using match vectors. After background subtraction, protein spot volumes were normalized against total spot volume of all proteins in a gel image. The proteins that significantly changed in abundance in response to treatments were identified subsequently by statistical analysis.

1.6. Mass spectrometry

Protein spots were excised from gels using a tissue puncher (Beecher Instruments, Prairie, WI, USA). Gel plugs were de-stained twice with 25 mmol L^{-1} ammonium bicarbonate in 50% acetonitrile, dehydrated with 100% acetonitrile and digested with 11 ng L^{-1} of trypsin (Promega, Madison, WI, USA) overnight at 37 °C. Digested proteins were extracted using elution buffer (0.1% trifluoroacetic acid (TFA/acetonitrile; 2:1)) and concentrated using a SpeedVac (Thermo Fisher Scientific, Waltham, MA, USA). The elution buffer containing the digested protein was mixed with 5 µL of matrix solution (0.2 mg L^{-1}-hydroxycyano cinnamic acid in acetonitrile) and spotted on an Anchorchip™ target plate (Bruker Daltonics Inc., Billerica, MA, USA). The spotted proteins were washed with 0.1% TFA and re-crystalized using an acetone/ethanol/0.1% TFA (6:3:1) mixture.

Peptide mass fingerprints (PMFs) were obtained on a matrix- assisted laser desorption ionization tandem time-of-flight (MALDI- TOF-TOF) mass spectrometer (Ultraflex II; Bruker Daltonics Inc., USA). We chose a minimum of six peptides to conduct tandem MS in order to obtain information about the b- and y-ions of the peptide sequence.

To analyze the peptide spectra, we used flexAnalysis (version 3.0; Bruker Daltonics Inc., USA) and applied the TopHat algorithm for baseline subtraction, the Savitzky–Golay analysis for smoothing (with: 0.2 m/z; number of cycles 1) and the SNAP algorithm to detect peaks (signal-to-noise ratio: 6 for MS and 1.5 for MS/MS). The charge state of the peptides was assumed to be +1. We used porcine trypsin for internal mass calibration. To identify proteins, we used Mascot (version 3.1; Matrix Science Inc., Boston, MA, USA) and combined PMFs and tandem mass spectra in a search against the *L. rubellus* database (http://badger.bio.ed.ac.uk/earthworm/; the site closed down since the data analysis has been completed). In the event of peptides matching to multiple members of a protein family, the presented protein was selected based on both the highest score and the highest number of matching peptides (minimum of two). Search parameters were set as follows: enzyme specificity: trypsin; one missed cleavage permitted. Variable modifications; carbamidomethylation of cysteine and oxidation of methionine. For tandem MS we set the precursor ion mass tolerance to 0.6 Da. The molecular weight search (MOWSE) score of > 32 indicated a significant hit.

1.7. Statistical analysis

Normalized spot volumes were analyzed with GraphPaD Prism 6.01. We conducted a one-way ANOVA to estimate difference between the exposure groups. For the one-way ANOVA a null distribution was generated using 1000 permutations to account for the unequal variance and non-normal distributions of the response variables, and a p-value of 0.05 was used. Proteins from the control and Cd-exposed group were compared using t-Tests (version 13.0, Systat Software GmbH Erkrath, Germany).

2. Energy phenotype Assay results

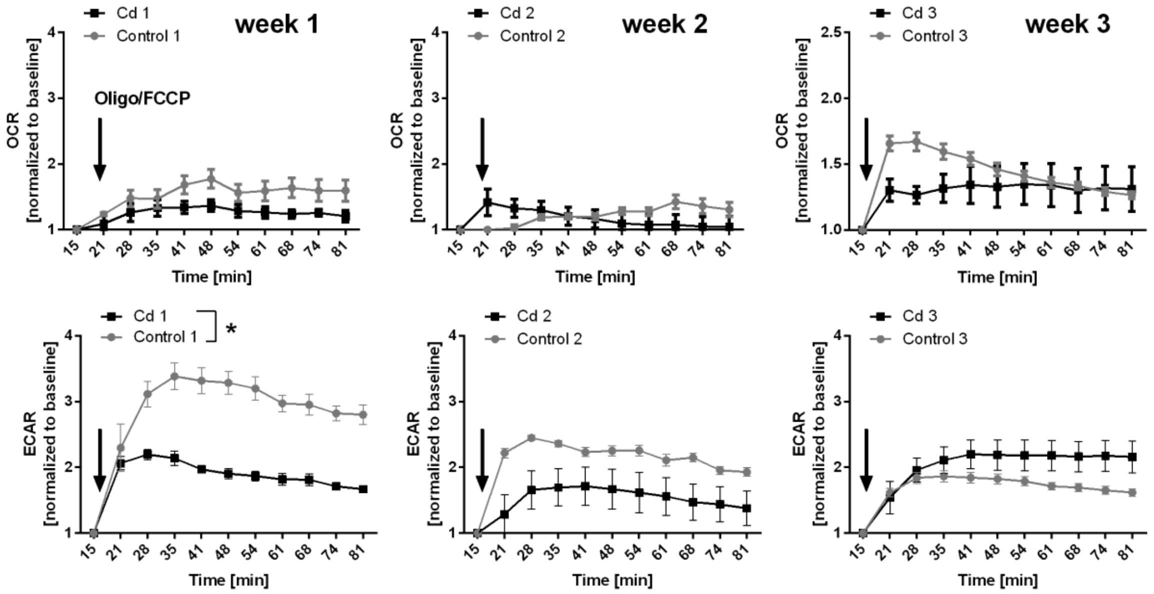

Figure S1. Energy phenotype assay using extracellular flux measurements of coelomocytes. *Two-Way ANOVA indicates significant differences at p≤0.05. Stressed phenotype refers to the state after addition of Oligo/FCCP.

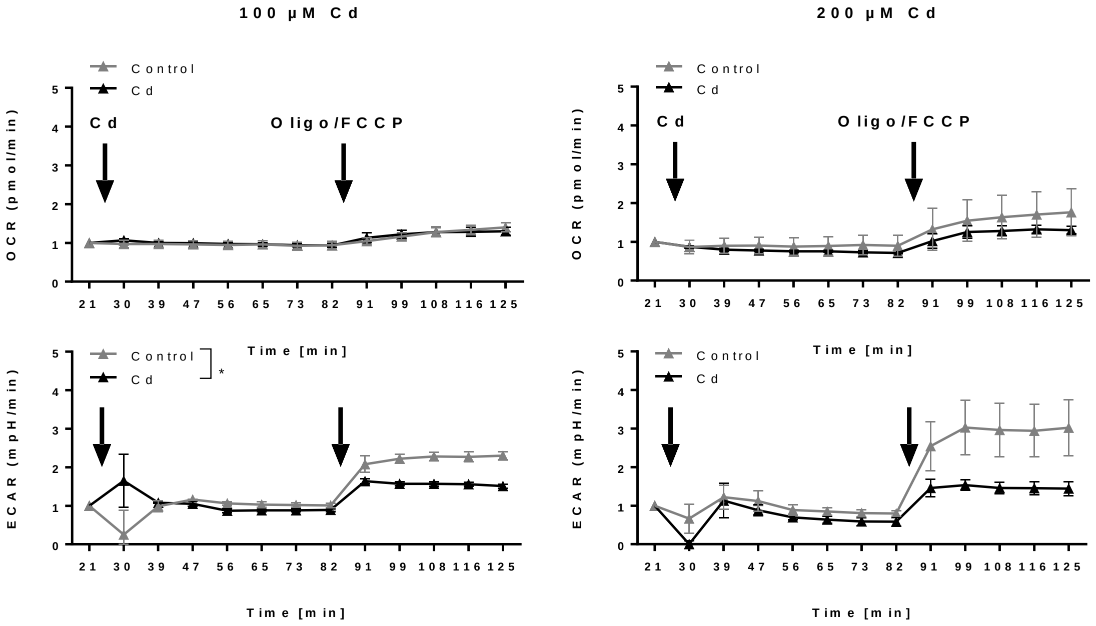

Figure S2. Energy phenotype assay using extracellular flux measurements from in vitro Cd exposed coelomocytes. *Two-Way ANOVA indicates significant differences at p≤0.05. Stressed phenotype refers to the state after addition of Oligo/FCCP.

3. Agilent Seahorse Assays

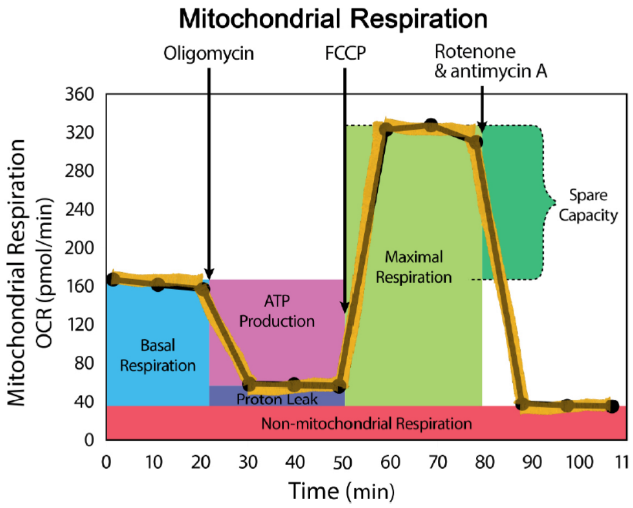

Figure S3. Agilent Seahorse XFp Cell Mito Stress Test profile of the key parameters of mitochondrial respiration. Sequential compound injections measure basal respiration, ATP production, proton leak, maximal respiration, spare respiratory capacity, and non-mitochondrial respiration. Oligomycin is an inhibitor of ATP synthase. Carbonyl cyanide 4-(trifluoromethoxy) (FCCP) is an ionophor and uncoupling agent. Rotenone/Antimycin A (Rot/AA) is an inhibitor of electron chain complex I and II. Inhibition of mitochondrial function by Rotenone and Antimycin A (Rot/AA) enables calculation of mitochondrial-associated acidification.

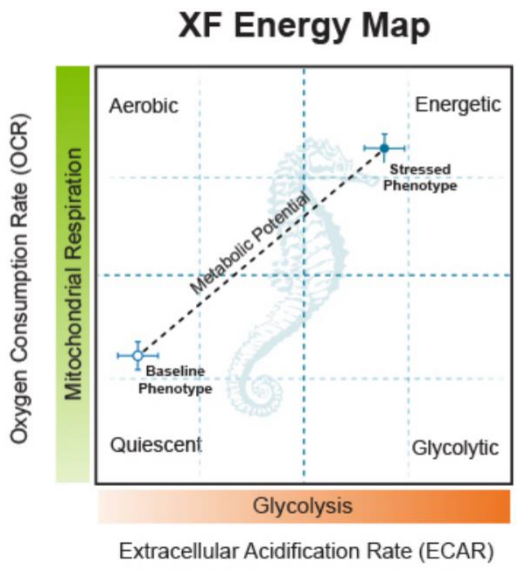

Figure S4. Agilent Seahorse XF Cell Energy Phenotype Profile. The relative utilization of the two energy pathways of a cell population is determined under both baseline (Baseline Phenotype) and stressed (Stressed Phenotype) conditions. The response to an induced energy demand is their Metabolic Potential. The stressed phenotype is induced by injection of oligomycin and Carbonyl cyanide 4-(trifluoromethoxy) (FCCP). Oligomycin is an inhibitor of ATP synthase; Carbonyl cyanide 4-(trifluoromethoxy) (FCCP) is a ionophor and uncoupling agent.

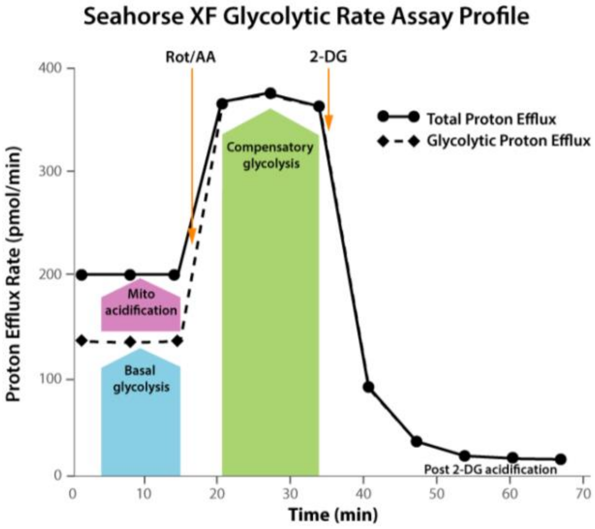

Figure S5. Agilent Seahorse XF Glycolytic rate assay profile. Proton efflux from live cells comprises both glycolytic and mitochondrial-derived acidification. Inhibition of mitochondrial function by Rotenone and Antimycin A (Rot/AA) enables calculation of mitochondrial-associated acidification. Subtraction of mitochondrial acidification to Total Proton Efflux Rate results in Glycolytic Proton Efflux Rate. Rotenone/Antimycin A (Rot/AA) is an inhibitor of electron chain complex I and II. 2-deoxy-glucose (2-DG) presents a glucose molecule that cannot be metabolized - it competitively inhibits the production of glucose-6-PO4 from glucose.
